# Biliary Dyskinesia as a Rare Presentation of Metastatic Breast Carcinoma of the Gallbladder: A Case Report

**DOI:** 10.1155/2011/806570

**Published:** 2011-09-21

**Authors:** A. Markelov, H. Taheri, K. Vunnamadala, G. Ibrahim

**Affiliations:** Department of Surgery, Easton Hospital, 250 S 21th Street, Easton, PA 18042, USA

## Abstract

*Background*. Breast carcinoma is the most common malignancy in women worldwide. It is most commonly associated with metastases to the liver, lung, bone, and the brain. Invasive lobular carcinoma is a less common pathology with slightly higher metastases to the upper gastrointestinal tract. Invasive lobular carcinoma metastasis to the gallbladder is extremely rare. *Method*. In this paper we are presenting a case of a 67-year-old female with metastases of invasive lobular breast cancer to the gallbladder six years after her therapy. *Conclusion*. This case clearly signifies the nature of the micrometastatic foci of the invasive lobular carcinoma even many years after a successful treatment.

## 1. Case Report

A 67-year-old female was seen in the clinic complaining of a two-week history of right nipple inversion. A mass was discovered following breast examination which wasbiopsied—pathology revealed infiltrative lobular carcinoma. The patient subsequently underwent a right modified radical mastectomy with level three lymph node dissections. Pathological studies revealed a 7 cm extensive invasive lobular carcinoma with some foci of in situ ductal carcinoma. 10/16 lymph nodes were also positive. The cells were strongly estrogen receptor positive, with greater than 20% being Ki-67 antigen positive. Staining was negative for human epidermal growth factor Receptor 2 (HER2/neu) marker. The patient successfully finished her chemotherapy and radiation treatments with continuous aromatase inhibitor therapy. Six years later, she developed symptoms of nausea accompanying a 20-pound weight loss over a period of two months. Subsequent workup with Hepatobiliary Iminodiacetic Acid scan revealed gallbladder dyskinesia. The patient underwent an uncomplicated laparoscopic cholecystectomy. Cytological examination of the gallbladder was significant for findings of foci of tumor with a single file arrangement present outside the muscularis propria and some tumor cells within the muscularis propria. There were estrogen and progesterone positive receptors with more than 10% Ki-67 antigen positive and HER2/neu negative marker. These findings were significant for metastatic lobular carcinoma of the breast.

## 2. Discussion

**Figure 1:**
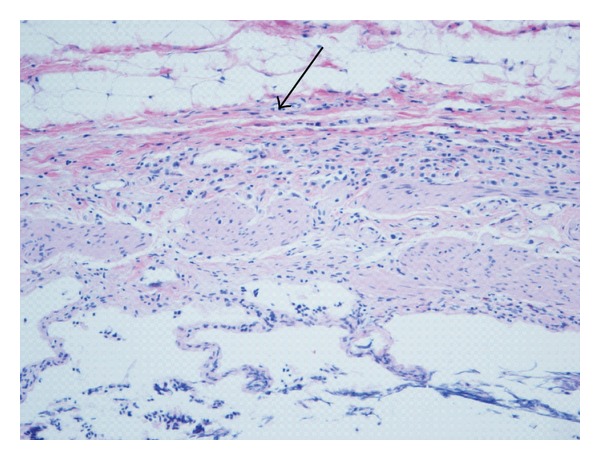
Invasion of the muscularis propria of the gallbladder by metastatic lobular carcinoma of the breast.

There is well-known evidence of breast cancer's metastatic potential with contiguous, lymphatic, and hematogenous spread. Common sites of metastasis include bone, lungs, and the liver [1, 2]. The central nervous system (CNS), endocrine organs (ovary, adrenal, pituitary), pericardium, abdominal cavity, and eye are infrequently involved organs [1]. Breast carcinoma metastasizing to the gallbladder is extremely rare and infrequently described. In one large autopsy series, metastases to the gallbladder were found only in 5.8% of cancer patients [3]. The tumor which is most likely to metastasize to the gallbladder is malignant melanoma (Figure 1) [4].

**Figure 2:**
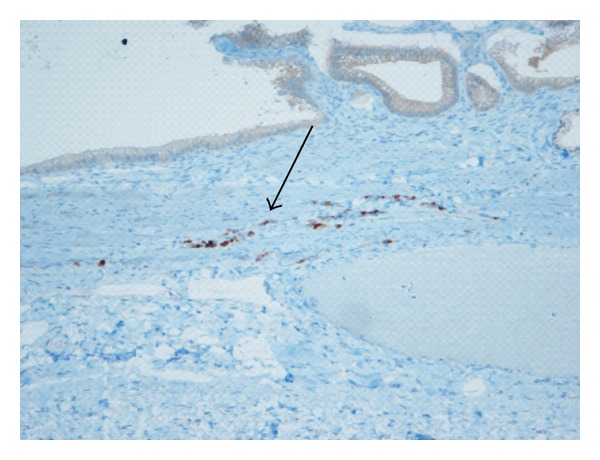
Estrogen positive cells in gallbladder wall on immunohistochemical staining.

Metastatic breast carcinoma involving the gallbladder or biliary tract presents with abdominal pain, symptoms of cholecystitis and obstructive jaundice [5–7]. Crawford et al. [8] reported a 73-year-old lady with breast carcinoma who developed cholecystitis and subsequently underwent cholecystectomy. The result of pathological examination was consistent with metastatic carcinoma from primary breast carcinoma. Similarly, Ferlicot et al. [9] reported two cases of metastatic breast cancer presenting as cholecystitis. Both patients had undergone mastectomy years earlier. Pathologic evaluation of the gallbladder specimen revealed metastatic infiltrating ductal carcinoma in one patient and infiltrating lobular carcinoma in the other (Figure 2 and Table 1) [9].

In our patient, we saw that the lobular carcinoma was accompanied by metastatic spread. Lobular carcinomas show a preference to gynecologic organs, peritoneum-retroperitoneum, and gastrointestinal system, including the gallbladder [10].

## 3. Conclusion

This is an extremely rare case of invasive lobular carcinoma with metastases to the gallbladder. This case clearly signifies the importance of the micrometastatic foci of the invasive lobular carcinoma many years after a successful treatment.

## Figures and Tables

**Table 1:** Feature of cases with metastases of breast carcinoma to gallbladder.

Reference	Type of breast cancer	Presentation	Diagnosis	Treatment	Outcome
Boari et al.	Invasive lobular carcinoma Invasive ductal carcinoma	RUQ pain, acute cholecystitis	RUQ US showed gallbladder mass and stones Carcinoma confirmed by pathology	Laparoscopic cholecystectomy	Cured
Zagouri et al.	Invasive lobular carcinoma grade II Invasive ductal carcinoma grade I	Intermittent RUQ pain	RUQ US showed stones Carcinoma confirmed by pathology	Laparoscopic cholecystectomy	Cured
Shah et al.	Not specified	Altered mental status	RUQ US, CT abdomen, paracentesis	Exploratory laparotomy	Expired POD no. 5
Crawford et al.	Infiltrating ductal carcinoma	Upper abdominal pain with nausea for 3 weeks	SBFT, RUQ US, oral cholecystogram	Laparoscopic cholecystectomy converted to open	One year s/p cholecystectomy
	Infiltrating lobular carcinoma	Upper abdominal postprandial pain for 6 months	RUQ US, oral cholecystogram, HIDA scan Carcinoma confirmed by pathology	Laparoscopic cholecystectomy converted to open	Expired 3 years later from disseminated metastases
Beaver et al.	Not specified	RUQ pain, nausea and vomiting (acute cholecystitis)	RUQ US Carcinoma confirmed by pathology	Cholecystectomy combined with chemotherapy (5FU, methotrexate, vincristine)	Cured
Case report	Invasive lobular carcinoma with foci of DCIS	Nausea and weight loss for 2 months	HIDA scan showed biliary dyskinesia Carcinoma confirmed by pathology	Laparoscopic cholecystectomy	Cured
